# Deep learning-based stress detection from RR intervals in major depressive disorder, panic disorder, and healthy individuals

**DOI:** 10.3389/fpsyt.2025.1672260

**Published:** 2025-09-25

**Authors:** Kyung Hyun Lee, Chul-Hyun Cho, Ah Young Kim, Hong Jin Jeon, Sangwon Byun

**Affiliations:** ^1^ Department of Electronics Engineering, Incheon National University, Incheon, Republic of Korea; ^2^ Department of Psychiatry, Korea University College of Medicine, Seoul, Republic of Korea; ^3^ Department of Biomedical Informatics, Korea University College of Medicine, Seoul, Republic of Korea; ^4^ Medical Information Research Section, Electronics and Telecommunications Research Institute, Daejeon, Republic of Korea; ^5^ Department of Psychiatry, Depression Center, Samsung Medical Center, Sungkyunkwan University School of Medicine, Seoul, Republic of Korea; ^6^ Meditrix Co., Ltd., Seoul, Republic of Korea

**Keywords:** RR intervals, major depressive disorder, panic disorder, stress detection, deep learning, machine learning, autonomic nervous system, physiological signals

## Abstract

**Background:**

Stress exacerbates major depressive disorder (MDD) and panic disorder (PD), highlighting the need for continuous stress quantification. Because stress modulates autonomic function, heart rate variability (HRV) is commonly studied for stress detection. However, conventional HRV pipelines require 5-min recordings and handcrafted features, limiting real-time use. We evaluated whether a one-dimensional (1D) residual network can identify acute cognitive stress directly from ultra-short RR interval (RRI) signals in MDD, PD, and healthy controls (HCs).

**Methods:**

One hundred forty-seven adults (MDD = 41, PD = 47, HC = 59) completed up to five lab visits over 12 weeks. At each visit, RRIs were recorded during a 5-min resting baseline and a 5-min mental-arithmetic stressor. A 1D ResNet34 classified baseline versus stress from raw RRIs using both 5-min segments and 1-min epochs. Group-specific models were compared with a combined model trained on pooled data. Generalized estimating equations tested group and phase effects on RRIs.

**Results:**

Stress shortened RRIs in every group, but less in patients with MDD and PD than in HC. Combined training outperformed group-specific training: for 5-min data, accuracies reached 0.866 (MDD), 0.865 (PD), and 0.897 (HC); 1-min accuracies were 0.788, 0.815, and 0.797, respectively.

**Conclusion:**

Deep learning on raw RRIs detects acute cognitive stress across psychiatric and healthy cohorts without feature engineering. Five-minute windows still yield the best performance, yet 1-min epochs still achieve accuracies of approximately 0.80, demonstrating feasibility for integration into real-time monitoring tools for relapse prevention and personalized care in psychiatry.

## 1 Introduction

Major depressive disorder (MDD) and anxiety disorders, including panic disorder (PD), affect more than 250 million and 300 million people worldwide, respectively, and are leading contributors to disability and diminished quality of life (1, 2). MDD is characterized by persistent low mood, anhedonia, and somatic symptoms (3, 4), whereas PD involves recurrent panic attacks and anticipatory anxiety that disrupt daily functioning (4, 5). Left untreated, both conditions can impair cognition and increase suicide risk (6–8).

Stress is an important psychosocial factor of these illnesses. Previous studies show that both chronic exposure to stressors and acute stressful events increase the likelihood of onset, relapse, and a more refractory disease course in MDD and PD (9–15). Consequently, technologies capable of continuously quantifying the severity and duration of stress at the individual level are needed to enhance treatment and long−term management. In response, research increasingly utilizes wearable sensors to detect stress through physiological signals, demonstrating the feasibility of unobtrusive stress monitoring in daily life (16).

Heart rate variability (HRV)—the variability in successive RR intervals (RRIs)—is a widely used proxy for autonomic nervous system (ANS) responses to stress (17–19). Conventional pipelines typically compute time, frequency, and non-linear features from 5-min ECG segments (20) and, in healthy samples, machine-learning models using these features often exceed 0.80 accuracy (21, 22). Shorter windows (1 min) can retain acceptable signals for classification, although longer windows may still be preferred when greater robustness is required (23, 24). Nonetheless, feature-based HRV pipelines depend on parameter choices and their reliance on 5-min segments limits high−resolution, real−time use.

Despite extensive work in healthy cohorts, automated stress detection in psychiatric populations remains limited. These disorders show autonomic dysregulation—reduced baseline vagal tone and altered sympathetic reactivity (25–30)—which can complicate classification. In our previous study, classical classifiers using 20 HRV features from 5-min windows during a stress-relaxation protocol achieved overall accuracies of 0.94–0.96, with lower performance in patients with MDD and PD than in healthy controls (HCs) (31). Yet this approach required uninterrupted 5-min windows and handcrafted features, motivating a raw-signal strategy with shorter inputs.

Deep neural networks for one-dimensional (1D) time series can learn discriminative representations directly from RRIs, removing the need for feature engineering. Prior work in healthy participants reported successful performance using 10–30 s RRI windows (32) or convolutional representations (33), but clinically diagnosed MDD or PD populations have been underrepresented.

We address this gap by evaluating end-to-end stress detection from raw RRIs in a clinically characterized cohort comprising MDD, PD, and HCs. We adapted ResNet34 to a 1D architecture and examined two window lengths: a conventional 5-min segment and an ultra-short 1-min epoch. The 1-min window balances feedback latency with performance and aligns with evidence that ultra-short HRV becomes more reliable at ≥ 60 s (34). We hypothesized that deep learning models trained on 1-min RRI segments would achieve accurate stress detection in both patient cohorts and HCs.

In summary, we proposed a deep-learning framework that (i) eliminates reliance on handcrafted HRV features, (ii) operates on ultra-short RRIs suitable for continuous wearable monitoring, and (iii) is validated across MDD, PD, and HC groups, thereby clarifying the utility of raw-RRI, end-to-end models in psychiatric populations.

## 2 Methods

### 2.1 Participants and study design

This study was part of a larger investigation examining changes in clinical symptoms and inflammatory biomarkers over 12 weeks to capture treatment effects (35). As these methods have been described in detail in our previous publication, we only briefly introduce them here (35). A total of 147 participants were included in the study: 41 patients with MDD, 47 patients with PD, and 59 HCs. All patients were recruited at the Samsung Medical Center in Seoul, Korea, between December 2015 and January 2017. The diagnosis of MDD and PD followed the Diagnostic and Statistical Manual of Mental Disorders, Fifth Edition (DSM-5) criteria (4), and was conducted by a senior psychiatrist. Exclusion criteria were pregnancy, history of substance or alcohol abuse, head injury, high suicide risk, personality disorders, severe physical illnesses, and use of long-acting medications. Throughout the 12-week experiment, all patients received standard pharmacotherapy. Participants’ acute-episode or stable-treatment status was not prospectively labeled at enrollment. HCs with no history of psychiatric issues or family history of mood disorders were recruited via general advertisements. The study protocol was approved by the Ethics Committee of the Samsung Medical Center (No. 2015-07-151), and all participants provided written informed consent. Each participant received $50 as compensation.

Each participant underwent a 12-week study with five scheduled lab visits at baseline and 2, 4, 8, and 12 weeks. At the initial and final visits, demographic information (e.g., age and sex) was collected and clinical evaluations, including Hamilton Depression (HAMD), Hamilton Anxiety (HAMA), and Panic Disorder Severity (PDSS) scales, were performed (36–38). Body mass index (BMI) was also measured given its known influence on ANS response (39).

### 2.2 Experimental protocol

**Figure 1:**
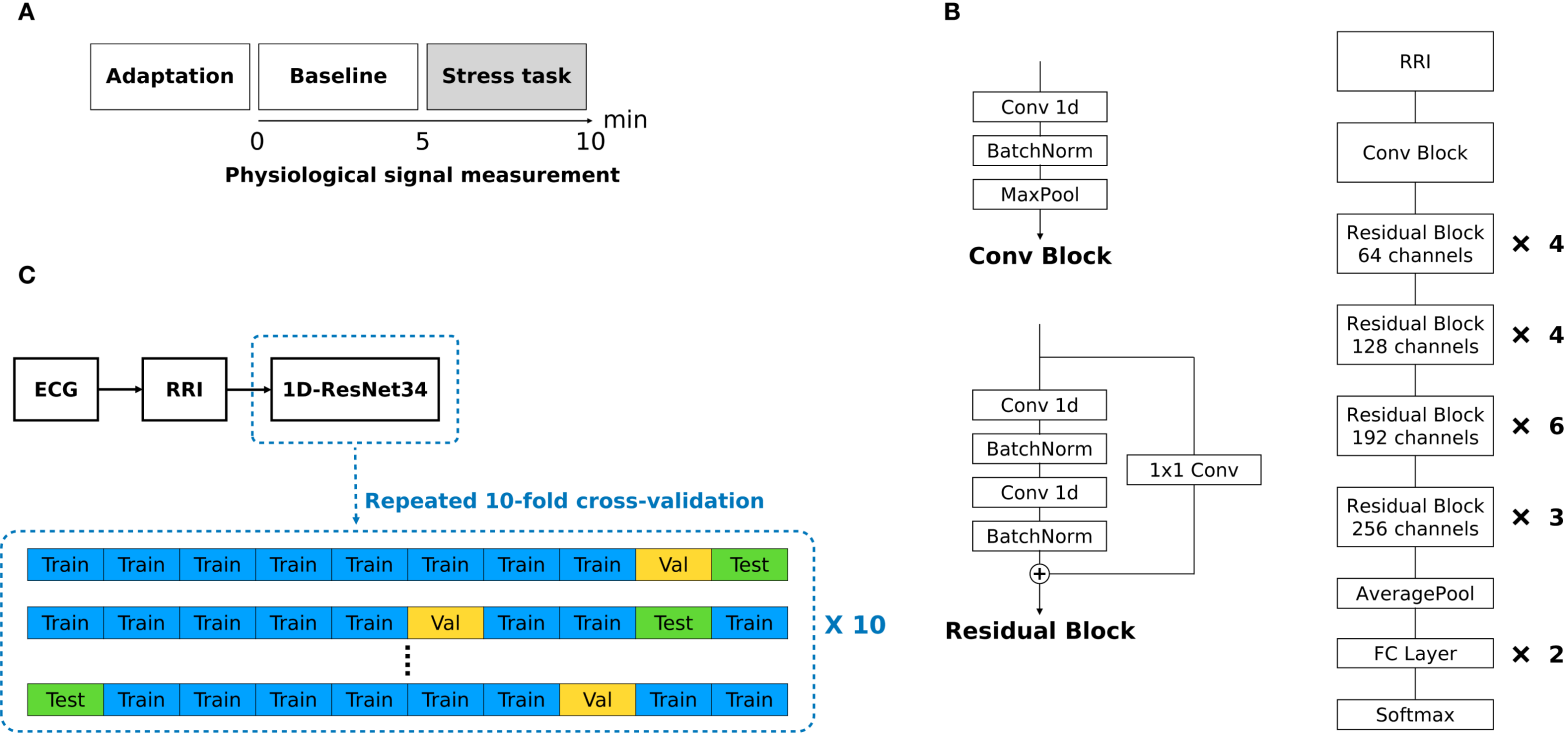
**(A)** Experimental protocol. **(B)** Overall architecture of modified 1D ResNet34. **(C)** Overview of data processing.

The original protocol comprised five phases. In this study we analyzed only the 5-min resting baseline and the 5-min mental-arithmetic stress (MAT) phases to detect stress-induced changes in continuously measured RRIs (**Figure 1A**). During baseline, participants rested quietly; during MAT, they performed serial-7 subtraction from 500 with error correction, a validated cognitive stressor known to modulate autonomic indices (40–44). The remaining recovery, relaxation, and final rest phases are described in the **Supplementary Methods**. Sessions were conducted by trained investigators in the clinical laboratory.

### 2.3 RRI measurement

All measurements were conducted during working hours to reduce variability associated with time of day, mood, and rest (45–47). Electrocardiogram (ECG) signals were captured using the ProComp Infiniti system (SA7500, Thought Technology, Montreal, Canada) at a sampling rate of 256 Hz (20). RRIs were then extracted and processed in Kubios HRV Premium (48, 49) using an in-house developed QRS detection algorithm based on the Pan-Tompkins method. Each RRI series was resampled to an equidistant 4 Hz data using cubic-spline interpolation. **Supplementary Figure S1** presents an example of the RRI values measured during the baseline and stress phases. Full measurement details are provided in the **Supplementary Methods**.

### 2.4 Statistical analyses

All analyses were performed using SPSS version 25 (SPSS Inc., Chicago, IL, USA) and Python version 3.11.4 (Python Software Foundation). One-way analysis of variance (ANOVA) was used for demographic and clinical variables across the MDD, PD, and HC groups, except for sex (chi-square test). For 5-min RRIs, we used generalized estimating equations (GEE) to estimate population-average phase and group effects after preliminary mixed-effects models indicated substantial within-subject autocorrelation. GEE is appropriate for correlated repeated measures and yields robust standard errors (50, 51). Fixed effects were phase (baseline, stress; baseline = reference), group (MDD, PD, HC; HC = reference), phase × group, and visit (1–5, categorical). Participants were treated as clusters, observations were ordered by visit and then phase, and an exchangeable working correlation was adopted because an AR (1) structure failed to converge when some clusters contained only two observations. To test whether the change from baseline to stress differed within each group, we computed phase-specific contrasts by summing the main phase effect with its interaction term for each group, and evaluated these contrasts using Wald z-statistics. Similarly, we then fitted a GEE to the 1-min RRI epochs—the last two minutes of the baseline (B4 and B5) and the first two minutes of the stress task (S1 and S2). The fixed-effects design was as follows: epoch (B4, B5, S1, and S2; S1 = reference) × group (MDD, PD, and HC; HC = reference) + visit (1–5), with participants as clusters. For each group, we obtained epoch-specific contrasts to determine whether the 1-min RRI during B4, B5, or S2 differed significantly from that during S1. A *P* value of < 0.05 was considered statistically significant.

### 2.5 Deep-learning architecture

We converted ResNet34 into a 1D architecture for raw RRI signals, as shown in **Figure 1B** (52, 53). ResNet was selected because of its strong time-series performance and prior success with RRI arrhythmia classification (54, 55). The model began with a convolutional block comprising a single 1D convolution, batch normalization, and max pooling. Each residual block contained three 1D convolutional layers and two batch-normalization layers, with an additional 1D convolution (kernel size = 1) as the shortcut connection. Gaussian error linear units (GELU) replaced ReLU activations throughout, and batch normalization preceded each activation to capture subtler non-linear patterns. The network processed fixed-length inputs of 1200 points for a 5-min RRI segment and 240 points for a 1-min RRI epoch, padding shorter sequences with zeros. **Supplementary Table S1** lists details of the model architecture. **Supplementary Figure S2** shows representative training and validation loss curves produced by the modified 1D ResNet34 classifier.

### 2.6 Performance evaluation and training strategy

Model performance was evaluated using 10× repeated 10-fold cross-validation (CV) (**Figure 1C**). To prevent cross-participant contamination (data leakage), splits were made at the participant level to ensure that no subject appeared in both the training and test sets. In each split, eight folds were used for training, one for validation, and one for testing. This process was repeated 10 times with different random seeds. We report accuracy, the area under the receiver operating characteristic curve (AUROC), sensitivity, and specificity as the mean ± standard deviation across repetitions. For 5-min RRIs, the classifier distinguished between baseline and stress (with stress being positive). For 1-min RRIs, we evaluated three binary tasks: B4 vs. B5 (B4 = positive), B5 vs. S1 (S1 = positive), and S1 vs. S2 (S2 = positive).

The two training strategies were compared. Separate models were trained and evaluated within each diagnostic cohort (MDD, PD, and HC) using only that group’s data. The combined models were trained on a pooled dataset comprising all the groups, after which the performance metrics were computed separately for each cohort in the test datasets. A full schedule (147 participants × 5 visits) would have produced 735 recordings, but missed visits left 650 baseline and 650 stress samples (181 MDD, 191 PD, and 278 HC) for a total of 1300 used in the analysis. Of the 147 participants, 110 completed five visits, 16 completed four, 4 completed three, 7 completed two, and 10 completed one. All attended visits included both phases; therefore, no RRI datasets were missing, and no imputation was required. Analyses used all available visit-level observations. Classifications were executed using Python.

## 3 Results

### 3.1 Demographic and clinical characteristics

**Supplementary Table S2** presents the demographic and clinical profiles of participants from the same cohort examined in our previous study (31). No significant differences in age, sex, or BMI were observed among the groups. As expected, participants with MDD and PD scored higher on the HAMD and HAMA than the controls, indicating more severe depressive and anxiety symptoms. The PDSS was the highest in the PD group, followed by the MDD group, and lowest in the HC group, consistent with diagnostic expectations.

### 3.2 RRI measurement results: stress-induced changes and between-group differences

**Figure 2:**
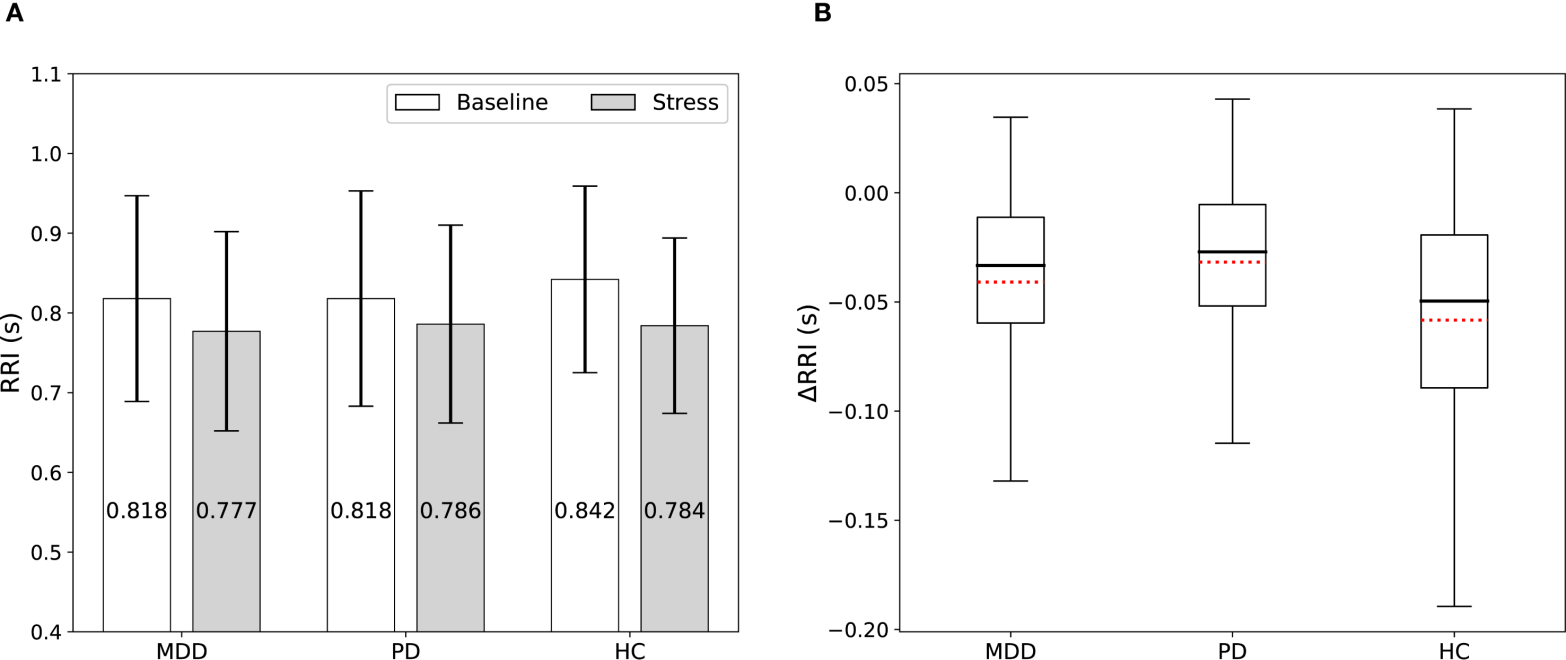
**(A)** RRI among the MDD, PD, and HC groups measured during the baseline and stress phases. Stress shortened RRI in all groups, but the magnitude of the reduction was smaller in patients. **(B)** Box plots display the ΔRRI. Red dotted lines indicate mean values.

**Figure 2A** and **Supplementary Table S3** show the RRI values measured for each group (MDD, PD, and HC) during the baseline and stress phases. Additionally, we presented within-subject changes in RRI (ΔRRI) from baseline to the stress task for each participant as presented in **Figure 2B** and **Supplementary Table S4**. The stress task elicited a significant decrease in mean RRI in every group (all *P* < 0.001), reflecting sympathetic activation with vagal (parasympathetic) withdrawal, which was observed as shorter RRI under stress (**Supplementary Table S5**). However, the magnitude of this reduction differed by group; it was significantly smaller in both the MDD group (*P* = 0.031) and the PD group (*P* < 0.001) than in HC, indicating that healthy participants exhibited the largest change from baseline (**Supplementary Table S5**).

### 3.3 Stress detection using 5-min RRIs

**Figure 3:**
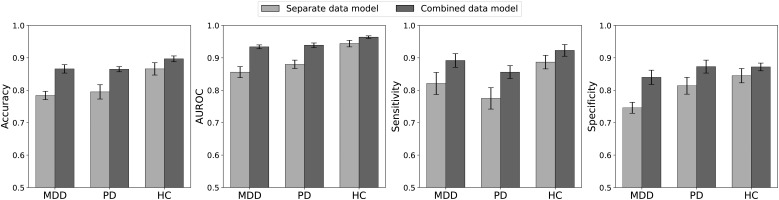
Performance measures for classifying the baseline and stress phases based on 5-min RRIs. Separate data models were trained and tested, each exclusively using the data from one specific patient group. For the combined data model, data from all groups were pooled for training, and the metrics were calculated separately for each patient group in the test dataset. The combined model outperformed the separate models across all groups, with HC generally achieving the highest accuracy.

Classification performance for distinguishing baseline from stress is summarized in **Figure 3** and **Supplementary Table S6**. When separate models were trained and tested exclusively on each diagnostic group, the highest accuracy was achieved by the HC group (0.866), followed by the PD (0.795) and MDD (0.784) groups. Notably, training a single “combined” model on data from all three groups improved performance for each group: HC accuracy rose to 0.897, while MDD and PD reached 0.866 and 0.865, respectively. Even within the combined model, HC consistently outperformed the clinical groups.

Analysis of the performance metrics indicates that the combined model generally outperformed the separate models for each group. Notably, across all these metrics, the HC group tended to outperform the other two clinical groups. The only exception was that, under the combined model, the specificity of the PD group was slightly higher than that of the HC group. In summary, despite being exposed to the same stress stimulus, the HC group achieved more accurate stress detection than the two clinical groups. Moreover, a model trained on pooled data from all groups produced better overall performance, underscoring the benefits of using a more diverse training set to enhance classification accuracy across diagnostic categories.

### 3.4 Stress detection using 1-min RRIs

**Figure 4:**
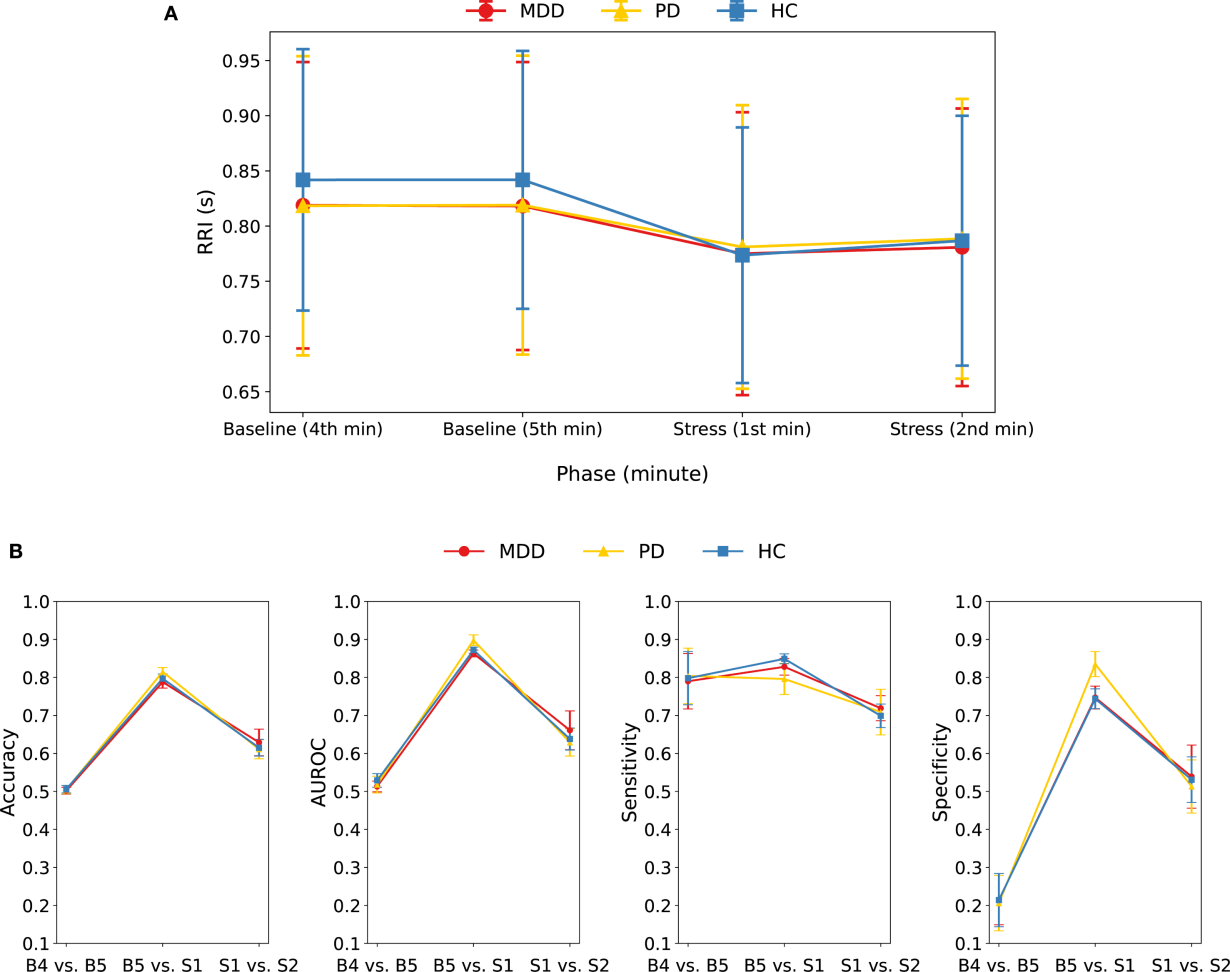
**(A)** Mean and standard deviation of RRI for each group during four consecutive 1-min epochs: the last two minutes of baseline (B4, B5) and the first two minutes of the stress task (S1, S2). RRI during S1 was lower than B4 and B5 in all groups; the decrease from B5 to S1 was smaller in MDD and PD than in HC, and RRI rebounded from S1 to S2 in HC and PD, but not in MDD. **(B)** Performance metrics of the combined model when classifying 1-min RRI epochs in three pairwise comparisons (B4 vs. B5, B5 vs. S1, S1 vs. S2) within each group.

We conducted an additional analysis in which the continuous RRI series was segmented into four non-overlapping 1-min epochs—the last two minutes of baseline (B4 and B5) and the first two minutes of the stress task (S1 and S2). **Figure 4A** and **Supplementary Table S7** show the RRI changes across the four 1-min epochs for each group. RRI during S1 was significantly lower than during either baseline epoch (B4 or B5) across all groups (all *P* < 0.001) (**Supplementary Table S8**). The B5-to-S1 decrease differed across groups; both patient groups—MDD (*P* = 0.004) and PD (*P* < 0.001)—showed a smaller decrease than HCs, consistent with the 5-min phase analysis. RRI rebounded from S1 to S2 in HC (*P* < 0.001) and PD (*P* = 0.001), but not in MDD, and this S1-to-S2 change did not differ between HC and PD patients (**Supplementary Table S8**).

We evaluated three binary classification tasks: B4 vs. B5, B5 vs. S1, and S1 vs. S2, within each group by applying the combined model to 1-min RRI segments (**Figure 4B**, **Supplementary Table S9**). Baseline minutes (B4 vs. B5) were indistinguishable (accuracy = ~0.50), whereas the baseline-to-stress change (B5 vs. S1) was detected with high accuracy (0.79–0.82). Discriminating the two stress minutes (S1 vs. S2) produced intermediate performance (accuracy = 0.61–0.63), indicating additional but less pronounced autonomic change beyond the initial stress response. Collectively, these findings confirm that the transition from baseline to stress is readily detectable within the first minute, whereas intra-baseline differences are negligible, and also that stress-epoch differentiation is modest.

A closer inspection of the B5 vs. S1 classification revealed that shortening the analysis window from 5-min segments to 1-min RRI epochs lowered overall performance: accuracy dropped by 0.10 in the HC, 0.08 in the MDD, and 0.05 in the PD groups, respectively. In this 1-min analysis, accuracy was highest in the PD group, followed by the HC and then MDD groups, whereas the 5-min model had HC at the top. Notably, although HC showed the largest mean 1-min RRI drop from baseline to stress, its accuracy still trailed PD’s. The PD group also achieved the highest specificity, indicating that baseline epochs were misclassified as stress less often than in the other groups. The shift in accuracy between 5- and 1-min inputs likely reflects group-specific temporal dynamics, whereby window length interacts with each group’s reactivity time course, explaining the change in ranking. For an overall comparison of the 1-min and 5-min models, group-specific ROC curves (MDD, PD, HC) from the combined model are shown in **Supplementary Figure S3**.

## 4 Discussion

This study investigated whether a 1D residual neural network could directly identify acute cognitive stress from raw RRI sequences in patients with MDD, PD, and HC. When the 5-min RRIs were analyzed with a model trained on the pooled dataset, accuracies reached 0.866 in MDD, 0.865 in PD, and 0.897 in HCs. Using 1-min windows lowered performance, yet accuracy remained at 0.788, 0.815, and 0.797 in the same groups. Taken together, these results demonstrate that raw signal models can approach 80% accuracy for stress classification—even in psychiatric cohorts—using recording periods as short as 1 min and without reliance on handcrafted HRV features.

All three diagnostic groups demonstrated a significant reduction in the RRI during the MAT, indicating that the protocol effectively triggered sympathetic and vagal withdrawal responses. Notably, the extent of RRI reduction during the stress phase was less pronounced in individuals with MDD or PD than in HCs. This finding aligns with existing literature suggesting altered autonomic reactivity in psychiatric disorders (28–30, 56). Importantly, this pattern of results was consistent across both the 5-min and 1-min windows, highlighting that significant clinical group differences can be detected even in ultra-short recordings.

The model performance reflected these physiological trends. For 5-min segments, the HC group—showing the largest RRI change—achieved the highest accuracy; MDD and PD, which displayed smaller ΔRRI, were classified less accurately. Pooling data for training across the groups improved the accuracy in every group, implying that a common representation of stress exists in raw RRIs that can be exploited through multi-cohort learning, even when absolute reactivity differs. In contrast, shortening the analysis window reduced accuracy more steeply in HCs than in patients: the HC reduction was roughly 0.10, compared with 0.05–0.08 in MDD and PD. This inversion suggests that the current ResNet model captures short-lived, patient-specific patterns in the RRI signal that remain detectable at 1-min scales, whereas a more pronounced but slower HC response is partially lost when only 1-min data are available.

We adopted a 1-min RRI window—the shortest duration considered reliable in ultra-short HRV research (34)—because no RRI-specific benchmark clearly defines how segment length affects stress-classification performance (32). In our study, shortening the input from 5-min to 1-min lowered accuracy by up to 0.10, indicating a length-performance trade-off. Windows shorter than 1-min will probably decrease accuracy further, but this needs confirmation. Future work should test sub-minute windows while also verifying that the stress protocol remains sufficiently potent at such short time scales.

Within the 5-min stress phase, RRI increased modestly between the first and second minutes (S1 vs. S2) in HCs and patients with PD, but not in patients with MDD. The lack of an RRI rebound from S1 to S2 in MDD is compatible with the impaired autonomic adaptability reported in depression and may reflect slower recovery. Although accuracy for classifying S1 and S2 was only 0.61–0.63, these results suggest that the network was able to detect physiologically meaningful variation within the continuous stress period. Participants may have experienced the greatest sympathetic activation during the initial minute of the MAT, followed by partial autonomic adaptation as subtraction continued. The resulting attenuation of arousal would manifest as a rebound in RRI, which the deep-learning model captured, despite the small change in RRI. As the current protocol imposed a uniform 5-min stress block, the temporal evolution of stress-related RRIs could not be examined in finer detail. Future studies should employ stress paradigms that vary in duration or stimulus type—potentially replacing the MAT—to characterize minute-by-minute autonomic dynamics and evaluate whether sub-segments of the stress phase can be distinguished with higher precision.

Compared to studies focused solely on healthy individuals, our results provide a direct benchmark. Reviews in healthy volunteers typically report an accuracy of 0.80–0.95 with HRV features (21, 22) and approximately 0.85–0.90 with 10–30 s RRIs using deep learning (32, 33). Here, an end-to-end model trained directly on raw RRIs achieved 0.87–0.90 with 5-min inputs and 0.79–0.82 with 1-min inputs, while extending validation to clinically diagnosed MDD and PD. This highlights the novelty of raw signal stress detection in psychiatric cohorts and the benefit of pooled training.

The ability to detect stress from 1-min data intervals enhances the feasibility of real-world applications. Contemporary wearable devices are capable of acquiring such ultra-short cardiac segments with adequate signal fidelity (57), enabling the implementation of a sliding window approach to compute stress probabilities in near-real time. This is particularly beneficial for psychiatric patients, who often experience exacerbations of stress-related symptoms. For example, these tools could be integrated into practice to provide continuous monitoring for patients at high risk of relapse, enabling timely intervention. Furthermore, objective stress data could assist clinicians in personalizing pharmacological therapy and tracking treatment efficacy. However, continuous stress monitoring in psychiatric care carries ethical and practical considerations. Issues such as patient acceptability, the risk of over-medicalization from misinterpreting data, and data privacy should be carefully addressed before these tools can be responsibly integrated into clinical care.

### 4.1 Limitations

All patients received pharmacotherapy, which may modulate autonomic tone and partially alter stress responses, thereby influencing classification. While antidepressants can affect HRV, the evidence is mixed (58, 59). We also lacked prospective stratification by acute vs. stable clinical status and by treatment response, both of which could influence autonomic reactivity and HRV-based stress responses. Future studies should incorporate status- and response-based analyses. Sample size also limits generalization, particularly for MDD. We did not stratify model performance by sex or age; future larger cohorts should assess the effects of subgroups.

The MAT is an artificial laboratory task; performance in naturalistic settings, where stressors are diverse and confounded by physical activity, remains to be tested. Only RRI signals were analyzed. Fusion with electrodermal activity (EDA) or accelerometry may improve robustness, particularly when motion artifacts are present. Finally, although ResNet34 performed well, alternative sequence models were not evaluated and could yield further gains.

Future research should assess the model’s generalizability in ambulatory settings that involve free-living stressors and physical activity. Adaptive windowing strategies may further improve real-time performance, whereas multimodal fusion—combining RRI with EDA or other physiological signals—could enhance classification accuracy (60–62). Clinically, longitudinal studies that relate daily stress estimates to symptom trajectories and treatment responses are needed to determine whether RRI-based monitoring translates into better patient outcomes.

## 5 Conclusion

Deep learning applied to raw RRIs detects acute cognitive stress in healthy individuals and patients with MDD or PD. The method effectively obviates engineered HRV features and functions on 1-min windows, a duration compatible with contemporary wearable devices. Although 5-min segments still yield the highest accuracy, the modest loss in performance observed with 1-min windows is outweighed by the gains in temporal resolution and real-world applicability. These findings support the integration of raw-signal, end-to-end models into mobile psychiatry with the goal of delivering objective stress assessments.

## Data Availability

The datasets presented in this article are not readily available because of privacy restrictions. Requests to access the datasets should be directed to Sangwon Byun, swbyun@inu.ac.kr.
