# Supplementary material for: Deep learning-based stress detection from RR intervals in major depressive disorder, panic disorder, and healthy individuals
Source: Front Psychiatry. 2025 Sep 25;16:1672260. doi: 10.3389/fpsyt.2025.1672260 (PMC12508820; doi:10.3389/fpsyt.2025.1672260)
Supplement: Supplementary file 1 [file DataSheet1.pdf]

Supplementary Material

1 Supplementary Methods

1.1 Stress protocol and RRI measurement

Experiments were conducted in a sound-attenuated room maintained at 23 °C and 45–55% humidity. Participants were seated in an armchair and instructed to minimize movement and speech except when responding during the stress task. The protocol comprised five consecutive 5-min phases: resting baseline, MAT, rest (recovery from stress), relaxation (viewing 10 scenic images, 30 s each), and final rest (recovery from relaxation). During MAT, participants performed serial-7 subtraction from 500 aloud with error correction. If the sequence reached 3 before the 5-min interval ended, they restarted from 500.

ECG was recorded with the ProComp Infiniti SA7500 (Thought Technology) at 256 Hz using three forearm electrodes (T9306M, Thought Technology; negative on the right, positive and ground on the left) and a 60 Hz notch filter. RRIs were then extracted and processed in Kubios HRV Premium using an in-house-developed QRS detection algorithm based on the Pan-Tompkins method. Artifacts were detected using a threshold-based algorithm and subsequently verified by visual inspection; affected beats were corrected via piecewise cubic-spline interpolation. Each RRI series was resampled to an equidistant 4 Hz time series via cubic-spline interpolation to provide fixed-length inputs for the 1D CNN. A 4 Hz rate is sufficient to capture the HF band (0.15–0.40 Hz) while limiting computational load and preserving beat-to-beat trends, and it does not alter the ordering of RR events or the 1-min/5-min windowing. To ensure consistency, a single operator performed all data analyses from start to finish.

2 Supplementary Tables

Supplementary Table 1. Architecture of modified 1D ResNet34.

Block	Output shape for 5-min RRIs	Output shape for 1-min RRIs	Number of layers	Details
Conv	(64, 598)	(64, 118)	1	1×15, 64, stride 1, padding 7
			1	1×5 max pool, stride 2
Residual 1	(64, 598)	(64, 118)	4	$\begin{bmatrix} 1\times15, 64 \\ 1\times15, 64 \end{bmatrix}$, stride 1, padding 7
Residual 2	(128, 299)	(128, 59)	1	$\begin{bmatrix} 1\times15, 64 \\ 1\times15, 128 \end{bmatrix}$, stride 2, padding 7
			3	$\begin{bmatrix} 1\times15, 128 \\ 1\times15, 128 \end{bmatrix}$, stride 1, padding 7
Residual 3	(192, 150)	(192, 30)	1	$\begin{bmatrix} 1\times15, 128 \\ 1\times15, 192 \end{bmatrix}$, stride 2, padding 7
			5	$\begin{bmatrix} 1\times15, 192 \\ 1\times15, 192 \end{bmatrix}$, stride 1, padding 7
Residual 4	(256, 75)	(256, 15)	1	$\begin{bmatrix} 1\times15, 192 \\ 1\times15, 256 \end{bmatrix}$, stride 2, padding 7
			2	$\begin{bmatrix} 1\times15, 256 \\ 1\times15, 256 \end{bmatrix}$, stride 1, padding 7
Linear	(256)	(256)	1	Average pool (kernel_size = 75 for 5-min RRIs and 15 for 1-min RRIs)
	(2)	(2)	2	256-d fc, SoftMax

Batch normalization was applied after each convolutional layer, except for the residual connections.

Supplementary Table 2. Demographic and clinical characteristics of the MDD, PD, and HC groups. The data presented in this table were derived from the same patient cohort used in our previous study, with the relevant reference provided in the main text. Data are presented as means and standard deviations for continuous variables and as counts for categorical variables.

	MDD ($N = 41$)	PD ($N = 47$)	HC ($N = 59$)	F or χ^2 (P value)	Post-hoc
**Demographic data**					
Age (years)	42.02 ± 16.65	41.64 ± 14.39	38.49 ± 14.22	0.88 (.42)[a]	ns
Sex (M/F)	11/30	17/30	23/36	1.64 (.44)	ns
BMI (kg/m^2)	22.93 ± 3.41	23.29 ± 3.26	22.76 ± 3.17	0.35 (.71)[a]	ns
**Clinical data**					
HAMD	17.49 ± 7.07	13.87 ± 7.71	1.88 ± 1.75	143.26 (< 0.001)[b]	MDD, PD > HC[b]
HAMA	16.56 ± 8.46	15.11 ± 8.44	2.12 ± 2.22	103.53 (< 0.001)[b]	MDD, PD > HC[b]
PDSS	3.61 ± 5.74	12.53 ± 6.05	0.02 ± 0.13	107.16 (< 0.001)[b]	PD > MDD > HC[b]

MDD: major depressive disorder; PD: panic disorder; HC: healthy control; ns: not significant; BMI: body mass index; HAMD: Hamilton rating scale for depression; HAMA: Hamilton rating scale for anxiety; PDSS: panic disorder severity scale.

[a]Fisher's one-way ANOVA

[b]Welch's one-way ANOVA and Games-Howell post-hoc analysis

Supplementary Table 3. Comparison of 5-min RRI among the MDD, PD, and HC groups measured during the baseline and stress phases. Mean values were computed from all five visits.

Group	Baseline RRI (s)	Stress RRI (s)
MDD	0.818 ± 0.129	0.777 ± 0.125
PD	0.818 ± 0.135	0.786 ± 0.124
HC	0.842 ± 0.117	0.784 ± 0.110

MDD: major depressive disorder; PD: panic disorder; HC: healthy control.

Supplementary Table 4. Differences in 5-min RRI between baseline and stress calculated within the same participant during a single visit (ΔRRI). An RRI value measured during the baseline was subtracted from that during the stress task. Data are presented as the mean and standard deviation computed from all visits of all participants.

Group	ΔRRI (s)<br>Stress – Baseline
MDD	-0.041 ± 0.042
PD	-0.032 ± 0.040
HC	-0.058 ± 0.051

MDD: major depressive disorder; PD: panic disorder; HC: healthy control.

Supplementary Table 5. GEE results with phase-specific contrasts for 5-min RRIs.

**GEE coefficients**				
**Term**	**β (s)**	**Robust *SE* (s)**	***z***	***P* value**
Intercept (visit 1, baseline, HC)	0.825	0.014	59.88	< 0.001
Stress	–0.058	0.006	–10.56	< 0.001
MDD	–0.012	0.023	–0.52	0.601
PD	–0.023	0.022	–1.07	0.283
Visit 2	0.015	0.007	2.12	0.034
Visit 3	0.016	0.007	2.30	0.022
Visit 4	0.017	0.008	2.16	0.031
Visit 5	0.022	0.008	2.57	0.010
Stress × MDD	0.017	0.008	2.16	0.031
Stress × PD	0.027	0.007	3.63	< 0.001
**Within-group phase effect**				
**Group**	**ΔRRI (β, s)**	**Robust *SE* (s)**	***z***	***P* value**
MDD	–0.041	0.006	–6.95	< 0.001
PD	–0.032	0.005	–6.62	< 0.001

β: regression coefficient; robust SE: sandwich standard error; MDD: major depressive disorder; PD: panic disorder; HC: healthy control.

Supplementary Table 6. Performance measures for classifying baseline and stress.

Group	Accuracy	AUROC	Sensitivity	Specificity
**Separate data model**				
MDD	0.784 ± 0.013	0.856 ± 0.017	0.821 ± 0.034	0.746 ± 0.017
PD	0.795 ± 0.022	0.880 ± 0.013	0.775 ± 0.033	0.814 ± 0.026
HC	0.866 ± 0.019	0.944 ± 0.010	0.887 ± 0.021	0.845 ± 0.022
**Combined data model**				
MDD	0.866 ± 0.013	0.934 ± 0.006	0.892 ± 0.021	0.840 ± 0.022
PD	0.865 ± 0.008	0.939 ± 0.007	0.856 ± 0.020	0.873 ± 0.020
HC	0.897 ± 0.009	0.964 ± 0.004	0.923 ± 0.018	0.872 ± 0.012

Separate data models were trained and tested, each exclusively using the data from one specific patient group. For the combined data model, data from all groups were pooled for training, and the metrics were calculated separately for each patient group in the test dataset.

MDD: major depressive disorder; PD: panic disorder; HC: healthy control; AUROC: area under the receiver operating characteristic curve.

Supplementary Table 7. Mean and standard deviation of RRI for each group during four consecutive 1-min epochs: the last two minutes of baseline (B4, B5) and the first two minutes of the stress task (S1, S2).

Group	Baseline (fourth min)	Baseline (fifth min)	Stress (first min)	Stress (second min)
MDD	0.819 ± 0.130	0.818 ± 0.130	0.775 ± 0.128	0.781 ± 0.126
PD	0.819 ± 0.136	0.819 ± 0.136	0.781 ± 0.128	0.789 ± 0.127
HC	0.842 ± 0.117	0.842 ± 0.117	0.774 ± 0.116	0.787 ± 0.113

MDD: major depressive disorder; PD: panic disorder; HC: healthy control.

Supplementary Table 8. GEE results with epoch-specific contrasts for 1-min RRI.

**GEE coefficients**				
**Term**	**β (s)**	**Robust *SE* (s)**	***z***	***P* value**
Intercept (visit 1, S1, HC)	0.756	0.014	53.73	< 0.001
B4 (vs. S1)	0.068	0.006	10.59	< 0.001
B5 (vs. S1)	0.068	0.006	10.61	< 0.001
S2 (vs. S1)	0.013	0.003	4.40	< 0.001
MDD	0.015	0.023	0.68	0.499
PD	0.008	0.021	0.39	0.696
Visit 2	0.016	0.007	2.19	0.029
Visit 3	0.017	0.007	2.46	0.014
Visit 4	0.017	0.008	2.15	0.031
Visit 5	0.021	0.009	2.47	0.014
B4 × MDD	-0.024	0.009	-2.79	0.005
B5 × MDD	-0.025	0.009	-2.91	0.004
S2 × MDD	-0.007	0.004	-1.67	0.095
B4 × PD	-0.031	0.009	-3.52	< 0.001
B5 × PD	-0.030	0.009	-3.52	< 0.001
S2 × PD	-0.006	0.004	-1.50	0.134
**Within-group epoch effect**				
**Group and epoch**	**ΔRRI (β, s)**	**Robust *SE* (s)**	***z***	***P* value**
MDD, B4	0.044	0.006	7.39	< 0.001
MDD, B5	0.043	0.006	7.51	< 0.001
MDD, S2	0.006	0.003	1.78	0.074
PD, B4	0.037	0.006	6.25	< 0.001
PD, B5	0.038	0.006	6.61	< 0.001
PD, S2	0.007	0.002	3.24	0.001

β: regression coefficient; robust SE: sandwich standard error; MDD: major depressive disorder; PD: panic disorder; HC: healthy control.

Supplementary Table 9. Performance metrics of the combined model when classifying 1-min RRI epochs in three pairwise comparisons (B4 vs. B5, B5 vs. S1, S1 vs. S2) within each group.

Group	Accuracy	AUROC	Sensitivity	Specificity
**B4 vs. B5**				
MDD	0.502 ± 0.009	0.513 ± 0.014	0.790 ± 0.073	0.214 ± 0.065
PD	0.505 ± 0.011	0.518 ± 0.022	0.804 ± 0.073	0.206 ± 0.073
HC	0.506 ± 0.010	0.529 ± 0.018	0.798 ± 0.070	0.214 ± 0.070
**B5 vs. S1**				
MDD	0.788 ± 0.016	0.863 ± 0.009	0.828 ± 0.022	0.747 ± 0.03
PD	0.815 ± 0.011	0.898 ± 0.014	0.796 ± 0.041	0.835 ± 0.033
HC	0.797 ± 0.012	0.872 ± 0.008	0.849 ± 0.013	0.744 ± 0.026
**S1 vs. S2**				
MDD	0.629 ± 0.035	0.661 ± 0.051	0.719 ± 0.033	0.539 ± 0.083
PD	0.611 ± 0.025	0.629 ± 0.036	0.709 ± 0.06	0.513 ± 0.07
HC	0.615 ± 0.022	0.638 ± 0.029	0.699 ± 0.031	0.531 ± 0.06

For the combined data model, data from all groups were pooled for training and the metrics were calculated separately for each patient group in the test dataset.

MDD: major depressive disorder; PD: panic disorder; HC: healthy control; AUROC: area under the receiver operating characteristic curve.

3 Supplementary Figures

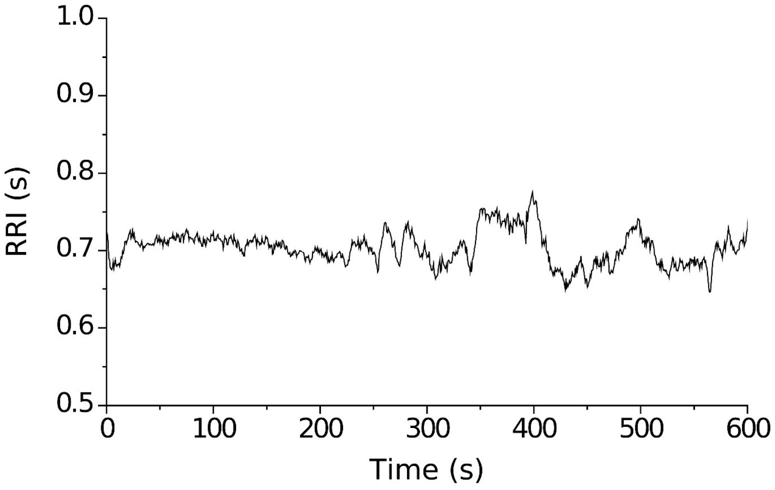

Supplementary Figure 1. Sample RRI data from the measurement.

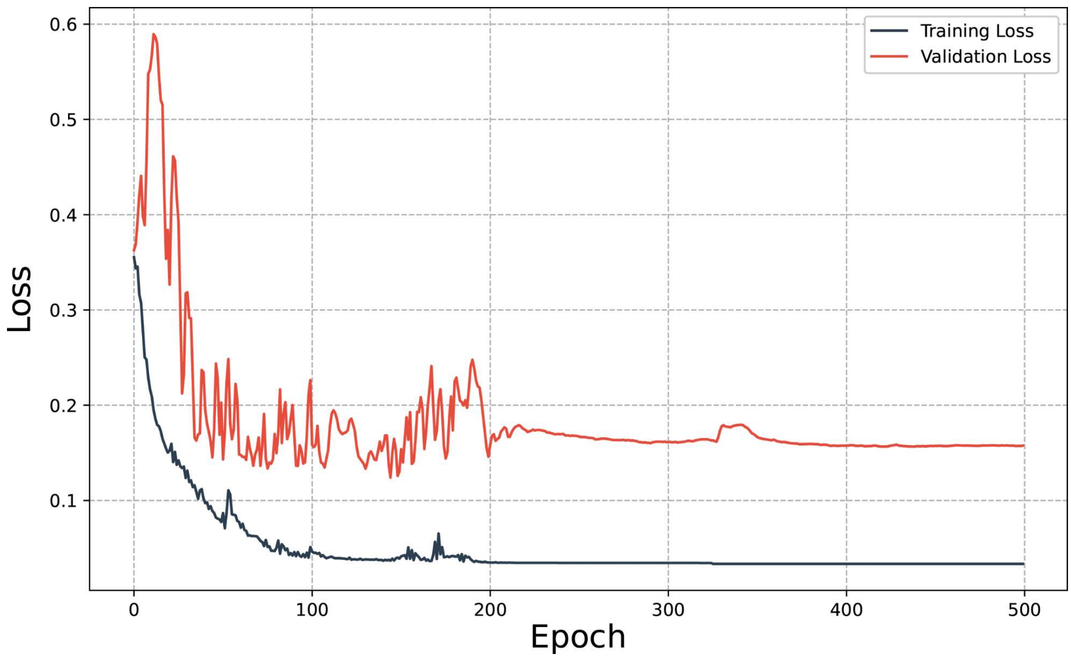

Supplementary Figure 2. Training and validation loss curves.

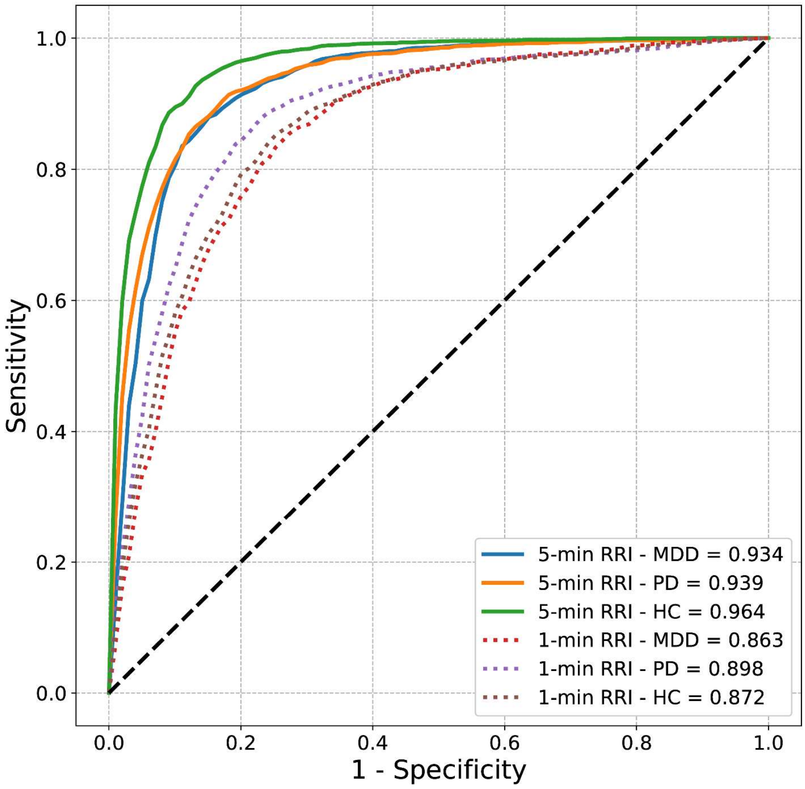

Supplementary Figure 3. ROC curves comparing the 1-min and 5-min RRI models across MDD, PD, and HC.
